# A randomised, open-labelstudy of insulin glargine or neutral protamine Hagedorn insulin in Chinese paediatric patients with type 1 diabetes mellitus

**DOI:** 10.1186/s12902-016-0146-2

**Published:** 2016-11-26

**Authors:** Min Liu, Zhiguang Zhou, Jinhua Yan, Pin Li, Wenhui Song, Junfen Fu, Xiaobo Chen, Weigang Zhao, Li Xi, Xiaoping Luo, Liang Sha, Xueyuan Deng, Chunxiu Gong

**Affiliations:** 1 Beijing Children’s Hospital, Capital Medical University, No.56 South Lishi Road, Xicheng District, Beijing, China; 2 The Second Xiangya Hospital of Central South University, Changsha, China; 3 The Third Affiliated Hospital, Sun Yat-sen University, Guangzhou, China; 4 Children’s Hospital of Shanghai, Shanghai Jiaotong University, Shanghai, China; 5 Children’s Hospital of Shanxi Province, Taiyuan, China; 6 The Children’s Hospital, Zhejiang University School of Medicine, Hangzhou, China; 7 Capital Institute of Pediatrics, Beijing, China; 8 Peking Union Medical College Hospital, Beijing, China; 9 Children’s Hospital Affiliated to Fudan University, Shanghai, China; 10 Tongji Hospital, Tongji Medical College, Huazhong University of Science & Technology, Wuhan, China; 11 Sanofi (China) Investment Co., Ltd., Shanghai, China

**Keywords:** Chinese paediatric patients, Insulin glargine, NPH insulin, Type 1 diabetes mellitus

## Abstract

**Background:**

We aimed to describe the safety and efficacy of insulin glargine in Chinese paediatric patients with type 1 diabetes mellitus (T1DM). Neutral protamine Hagedorn (NPH) insulin was the reference therapy.

**Methods:**

This open-label, randomised, Phase III study was conducted at 10 sites in China. Children aged ≥6 to <18 years with T1DM were randomised (2:1) to insulin glargine or NPH insulin asbasal insulinfor a 24-week treatment period. For all patients, insulin aspart was given as bolus insulin. The primary endpoint was absolute change in glycated haemoglobin(HbA1c) from baseline to Week 24. Secondary endpoints included the percentage of patients reaching HbA1c <7.5% (<58.5 mmol/mol), and safety. The study was registered at clinicaltrials.gov (NCT01223131).

**Results:**

In total,196 patients were screened, and 162 were randomised (107 and 55 patients were randomised to insulin glargine and NPH insulin, respectively). The mean ± SD of absolute change in HbA1c was–0.25 ± 1.68% (–2.69 ± 18.32 mmol/mol) in the insulin glargine group and –0.54 ± 1.67% (–5.55 ± 20.32 mmol/mol) in the NPH insulin group. At Week 24, 18.7 and 21.6% of patients in the insulin glargine and NPH insulin groups achieved HbA1c <7.5% (<58.5 mmol/mol). Both treatments were generally well tolerated. A numerically lower rate of symptomatic hypoglycaemia per patient year was observed for insulin glargine versus NPH insulin (24.3 ± 45.8 versus32.3 ± 43.2); severe hypoglycaemia was rare (<2%).

**Conclusions:**

Initiation of insulin glargine can aid Chinese paediatric patients with T1DM to safely reduce their HbA1c levels.

**Electronic supplementary material:**

The online version of this article (doi:10.1186/s12902-016-0146-2) contains supplementary material, which is available to authorized users.

## Background

The prevalence of type 1 diabetes mellitus (T1DM) among paediatric populations (aged <18 years) has been increasing globally over the last decade [1, 2]. Good blood glucose control can be achieved using multiple daily injections of basal and bolus insulin [3]. Several long-acting insulin analogues have been developed to provide a slow-release insulin that attempts to mimic the physiological action of natural basal insulinin healthy individuals [4]. Compared with intermediate-acting neutral protamine Hagedorn (NPH) insulin, long-acting insulin analogues have a relatively longer duration of action and a less pronounced peak insulin concentration [4].

Insulin glargine is an insulin analogue that was engineered to have a lower solubility at physiological pH than natural insulin, thereby facilitating a prolonged absorption following subcutaneous injection and providing asteady 24-h basal insulin supply [5, 6]. A meta-analysis of studies that were predominantly conducted in Europe and North America concluded that insulin glargine and NPH insulin have comparable efficacy in terms of HbA1c levels as well as broadly comparable safety profiles in paediatric patients with T1DM [7]. Nonetheless, insulin glargine is a once-daily injection, whereas NPH insulin dosing varies and can require multiple daily injections. Using once-daily insulin glargine to attain glycaemic control can thus reduce the total number of daily injections, which, in turn, can reduce lipodystrophy, injection-site pain and bruising, which is associated with multiple daily injections,particularly in younger children [8, 9]. Furthermore, insulin glargine has been associated with better control of fasting blood glucose (FBG) levels, lower incidence of hypoglycaemia,and nocturnal free insulin levels [10–13].

Insulin glargine is approved in Europe and the USA for use in both adults and paediatric patients with T1DM [5, 6]. However, in China, insulin glargine is approved for use only in adults with T1DM. To date, there have been relatively few reports of insulin glarginein paediatric patients with T1DM [7, 14, 15], and prior to the present study, there have been no reports regarding the efficacy and safety of insulin glargine in Chinese children with T1DM. Therefore, the purpose of the present study was to describe the safety and efficacy of once-daily insulin glargine over a period of 24 weeks in Chinese paediatric patients with T1DM.

## Methods

### Patients and study design

This was a 24-week,Phase III, randomised, open-label, parallel-group study. Paediatric patients aged ≥6 to <18 years with T1DM (duration ≥1 year) were eligible for enrolment. Other key exclusion criteria are described in the Additional file 1 (Methods). The study protocols and a subsequent protocol amendment were approved locally by independent ethics committees, and written informed consent was obtained from the parent or legal guardian of each patient. The study was performed in accordance with the Declaration of Helsinki, and registered at clinicaltrials.gov (NCT01223131).

Patients were centrally randomised in a block size of six and in a 2:1 ratio to receive insulin glargine (Lantus, Sanofi, Paris, France) by subcutaneous injection once daily at bedtime (20:00–22:00), or NPH insulin (Novolin N, Novo Nordisk, Copenhagen, Denmark) by subcutaneous injection either once daily at bedtime or twice daily: once before breakfast and once at bedtime. Investigators were allowed to select which of these two methods was more appropriate for their patients. Randomisation was conducted using an interactive voice response system and patients were stratified according to screening age (<12 years, ≥12 years) and screening HbA1c (<9% [<74.9 mmol/mol], ≥9% [≥74.9 mmol/mol]). A 2:1 randomisation ratio allowing a greater number of patients exposure to insulin glargine was used in order to help improve the precision of the safety assessment of insulin glargine in the patient population.

Insulin aspart (NovoRapid, Novo Nordisk) solution for injection was provided as the sole source of bolus insulin. Insulin dose was adjusted individually to maintain the desired degree of metabolic control without hypoglycaemia (Additional file 1).

The 24-week treatment period was preceded by a screening period of ≤2 weeks plus a 1-week run-in period, and followed by a 1-week post-treatment follow-up. Clinic consultations occurred at screening/run-in (Week –3 to –2 and Week –1), randomisation (Week 0), Weeks 1, 2, 4, 6, 8, 10, 12, 16, 20, 24 (end of treatment) and Week 25 (follow-up).

HbA1c was measured at screening, Week 0, Week 12, and Week 24 at a central laboratory using a high-performance liquid chromatography system (Variant™ II Hemoglobin Testing System, Bio-Rad, Hercules, CA, USA). The HbA1c reference values of the system were such that a HbA1c of <6% (<42.1 mmol/mol) was considered to be a non-diabetic level, a HbA1c of <7%(<53.0 mmol/mol) the target HbA1c level and a HbA1c of >8% (>63.9 mmol/mol) the need for action to lower HbA1c. The accuracy ranges (coefficients of variation) of the system were 0.59–0.90% within-run and 0.46–0.64% between-run. All blood glucose assessments, including FBG, nocturnal blood glucose, and self-monitored blood glucose (SMBG) profiles were self-monitored and collected for data analyses. FBG was measured in the morning on 6 consecutive days immediately preceding each clinic visit and on the day of the visit before insulin administration. Subjects established a four-point blood glucose profile (before each of the three main meals, and at bedtime) at least twice a week and as needed for glycaemic monitoring, and an eight-point glucose profile (at 3:00 a.m.; immediately before and 2 h after breakfast, lunch and dinner, and at bedtime) once a week. If the blood glucose measurement 2 h after dinner coincided with the bedtime measurement, reducing the eight-point SMBG to seven values was permitted. Plasma samples for pharmacokinetic analysis were obtained in the morning at Weeks 1, 2 and 4 from subjects in the insulin glargine group at selected sites, and samples were analysed at a central laboratory for concentrations of insulin glargine and its metabolites M1 and M2. Positivity for anti-glargine antibody (AGA) and anti-insulin antibody (AIA) was determined in a semi-quantitative manner by measurement of antibody binding to the iodinated insulin tracers 125I- insulin glargine and 125I-human insulin.

### Enrolment

Initially, it was planned to enrol 366 patients, with 244 patients randomised to insulin glargine and 122 to NPH insulin. However, recruitment of patients for the study was difficult due to the limited number of China Food and Drug Administration (CFDA)-accredited endocrinology sites and relatively low incidence of T1DM in China. Two years after enrolment of the first patient, a total of 108 patients were screened and 93 randomised, which constituted only 25% of the original enrolment target. A decision to amend the study protocol with a reduced enrolment target was made primarily to ensure the quality of the study data; 2 years into the study, the total recruitment period was estimated at >5 years, and over this timescale it would have been challenging to control the influence of factors such as changes in the standard of care on the study data. Therefore, the study protocol was amended to reduce the planned number of enrolled patients to 150, with 100 patients randomised to insulin glargine and 50 to NPH insulin (Additional file 1).

**Fig. 1:**
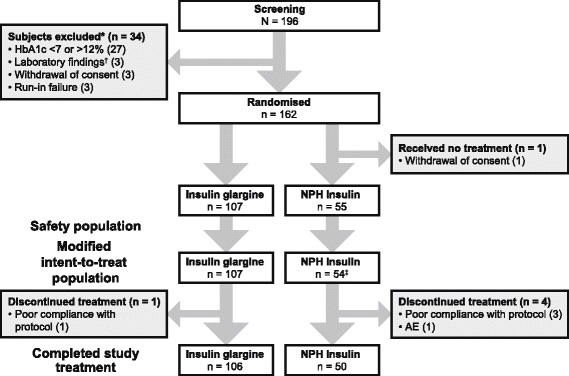
Patient flow diagram.*Patients could be excluded for more than one reason.^†^Serum creatinine > 177 μmol/l (2.0 mg/dl); serum alanine amino transferase or aspartate amino transferase > 3 times upper limit of normal for the patient’s age and gender; haemoglobin <10 g/dl (100 g/l) and/or neutrophils < 1500/mm^3^ (1.5 × 10^9^/l) and/or platelets < 100,000/mm^3^ (100 × 10^9^/l).^c^Threepatients had no post-baseline HbA1c assessment and were not included in the HbA1c analysis

Between February 2011 and March 2014, 196 patients were screened at 10 CFDA-accredited endocrinology centres in China. Of the 196, 34 patients were excluded and 162 enrolled (Fig. 1).

### Study outcomes

The primary endpoint of the study was absolute change in HbA1c from baseline to Week 24, and key secondary treatment efficacy endpoints were the percentage of patients reaching the International Society of Pediatric and Adolescent Diabetes (ISPAD)-recommended HbA1c target of <7.5% (<58.5 mmol/mol), and change in FBG, nocturnal blood glucose, 24-h blood glucose profile based on an eight-point SMBG profile measured using Performa blood glucose meters (Roche Diagnostics Gmbh, USA), and daily total insulin and basal insulin dose, from baseline to Week 24. SMBG profiles were used in posthoc analyses to assess glucose variability and the risk of hypoglycaemia and hyperglycaemia, which were defined by the low blood glucose risk index (LBGI) and the high blood glucose risk index (HBGI), respectively,and were calculated using previously described methods [16]. The insulin glargine pharmacokinetic profile was assessedat selected sites to rule out any tendency for accumulationin paediatric patients after repeated dosing, and safety parameters included AGA and AIA development, the incidence of hypoglycaemia, and adverse event monitoring. The classification of hypoglycaemia is described in the Additional file 1.

A posthoc analysis was performed to evaluate the percentage of patients with HbA1c 7.5–9% (58.5–74.9 mmol/mol) and > 9% (>74.9 mmol/mol) at Week 24, which indicates patients with acceptable but sub-optimal glycaemic control and high-risk patients, respectively, as defined by the Chinese Diabetes Society guidelines [17]. A posthoc analysis of the daily dose of basal and bolus insulin was also performed.

### Statistical methods

Based on the smaller-than-planned sample size, the protocol amendment made provision for descriptive statistics of the study endpoints, only. A statistical assessment of efficacy (not described in the protocol) was conducted as a sub-analysis. For this assessment, a modified intent-to-treat (mITT) population was used for statistical comparisons between treatment groups. The mITT was defined as all randomised patients who received at least one dose of study medication and for whom a baseline and at least one post-baseline assessment for any of the study endpoints were available. For patients with missing data at Week 24, the last observation carried forward (LOCF) approach was taken.

Differences between treatment groups for mean change in HbA1c, FBG, and daily total or basal insulin dose from baseline to Week 24 were assessed using an analysis of covariance (ANCOVA) model with treatment group, randomisation strata of screening age (<12 years, ≥12 years), randomisation strata of screening HbA1c (<9% [<74.9 mmol/mol], ≥9% [≥74.9 mmol/mol]) as fixed effects, and baseline value as covariate.

Differences between treatment groups in the number (%) of patients who achieved an HbA1c target of <7.5% (<58.5 mmol/mol) at Week 24 were assessed using the Cochran–Mantel–Haenszel method stratified by screening age strata (<12 years, ≥12 years) and screening HbA1c strata (<9% [<74.9 mmol/mol], ≥9% [≥74.9 mmol/mol]).

For the assessment of safety, data were described for a safety population, which was defined as all randomised patients who received at least one dose of study medication.

All continuous data were summarised using the number of observations available (n) and mean ± SD and median (quartile 1, quartile 3). Categorical data were summarised using the count and percentage. Further details are described in the Additional file 1.

## Results

### Patients

A total of 162 patients were randomised to receive insulin glargine (*n* = 107) or NPH insulin (*n* = 55). One patient randomised to NPH insulin withdrew consent; thus, the final mITT and safety populations for the insulin glargine and NPH insulin groups comprised 107 and 54 patients, respectively. Five patients who received at least one dose of study medication were discontinued from the study prematurely; one patient in the insulin glargine group and three patients in the NPH insulin group were discontinued due to poor compliance to the protocol; one patient in the NPH insulin group was discontinued due to a severe adverse event (SAE) of increased blood glucose. Therefore, a total of three patients in the NPH insulin group had no post-baseline HbA1c assessement and were not included in the statistical analysis for HbA1c.

Demographics and baseline characteristics were generally comparable between the two treatment groups (Additional file 2: Table S1). Compared with patients in the NPH insulin treatment group, a greater proportion of patients in the insulin glargine group were male (41.4% versus 35.2%). Patients in the insulin glargine group had a numerically lower mean baseline HbA1c level than those in the NPH insulin group (8.87 ± 1.21% [73.48 ± 13.27 mmol/mol] versus 9.12 ± 1.29% [76.16 ± 14.04 mmol/mol]). One patient in the NPH insulin treatment group had diabetic nephropathy (microalbuminuria).

### Efficacy

#### Primary endpoint

The mean absolute change in HbA1c level from baseline to Week 24 was–0.25 ± 1.68%(–2.69 ± 18.32 mmol/mol) for patients who received insulin glargine, and –0.54 ± 1.67% (–5.55 ± 20.32 mmol/mol) for those who received NPH insulin (Table 1). The median changes in HbA1c in the treatment groups were numerically similar. At Week 24, the mean HbA1c levels of the insulin glargine and NPH insulin treatment groups were 8.63 ± 1.54% (70.79 ± 16.81 mmol/mol) and 8.59 ± 1.79% (70.43 ± 19.60 mmol/mol), respectively.

**Table 1:** Summary of blood glucose control endpoints in the modified intention-to-treat analysis population with last observation carried forward for missing observations at Week 24

	Insulin glargine			NPH insulin		
	(*n* = 107)			(*n* = 54)		
	n	Mean ± SD	Median	n	Mean ± SD	Median
			(Q1, Q3)			(Q1, Q3)
HbA1c, % (mmol/mol)^a^						
Baseline	107	8.87 ± 1.21	8.90 (7.90, 9.70)	54	9.12 ± 1.29	8.90 (7.90, 10.20)
		(73.47 ± 13.27)	(73.77 [62.84, 81.97])		(76.16 ± 14.04)	(73.77 [63.11, 87.98])
Week 24	103	8.57 ± 1.47	(8.40 [7.70, 9.20])	50	8.60 ± 1.81	8.30 (7.60, 9.40)
		(70.13 ± 16.06)	(68.31 [61.20,76.50])		(70.49 ± 19.79)	(67.21 [59.56, 78.96])
Change^b^	103	−0.31 ± 1.56	−0.50 (–1.20, 0.60)	50	−0.57 ± 1.68	−0.40 (–1.20, 0.40)
		(–3.30 ± 19.52)	(–3.28 [–14.75, 7.65])		(–5.57 ± 21.51)	(–3.83 [-15.30, 5.19])
Week 24 (LOCF)^c^	107	8.63 ± 1.54	8.40 (7.70, 9.20)	51	8.59 ± 1.79	8.30 (7.60, 9.40)
		(70.79 ± 16.81)	(68.31 [61.20, 77.05])		(70.43 ± 19.60)	(67.21 [59.56, 78.69])
Change (LOCF)^b^	107	−0.25 ± 1.68	−0.50 (–1.20, 0.60)	51	−0.54 ± 1.67	−0.40 (–1.20, 0.40)
		(–2.69 ± 18.32)	(–5.46 [–13.11, 6.56])		(–5.55 ± 20.32)	(–3.28 [–15.30, 4.92])
LS Mean difference	0.16 (0.26)					
95% CI	−0.36 to 0.68					
HbA1c at Week 24, n (%)^a^						
<7.5% (<58.5 mmol/mol)^d^	107	20 (18.7)	--	51	11 (21.6)	--
7.5% to 9.0% (58.5–74.9 mmol/mol)	107	56 (52.3)	--	51	26 (51.0)	--
>9.0% (>74.9 mmol/mol)	107	31 (29.0)	--	51	14 (27.5)	--
Fasting blood glucose, mmol/L^e^						
Baseline	105	10.38 ± 3.38	10.08 (8.22, 12.24)	52	10.20 ± 2.75	10.59 (8.38, 11.88)
Week 24	96	9.45 ± 2.60	9.17 (7.56, 11.01)	44	10.89 ± 3.19	10.14 (8.51, 12.74)
Change^b^	94	−0.85 ± 3.73	−0.05 (–2.95, 1.24)	42	0.54 ± 3.34	0.49 (–1.45, 3.66)
Week 24 (LOCF)^c^	107	9.61 ± 2.63	9.30 (7.72, 11.59)	54	11.29 ± 3.35	10.81 (8.84, 13.00)
Change (LOCF)^b^	105	−0.76 ± 3.56	−0.05 (–2.47, 1.23)	52	1.07 ± 3.64	0.71 (–1.45, 3.86)
LS Mean difference	−1.69 (0.47)					
95% CI	−2.62 to –0.76					
Nocturnal blood glucose, mmol/L^f^						
Baseline	88	8.89 ± 4.47	8.00 (5.50, 11.80)	45	9.38 ± 4.81	8.10 (6.00, 12.00)
Week 24	89	9.44 ± 4.88	8.80 (5.60, 11.90)	42	8.88 ± 3.94	8.75 (5.80, 11.20)
Change^b^	75	0.42 ± 6.26	0.30 (–3.60, 4.60)	35	−0.14 ± 5.97	1.60 (–3.20, 3.70)
Week 24 (LOCF)^c^	106	9.44 ± 4.83	8.80 (5.60, 12.20)	54	9.56 ± 4.39	9.05 (6.20, 12.80)
Change (LOCF)^b^	88	0.59 ± 6.16	0.15 (–3.00, 3.95)	45	0.24 ± 5.80	1.60 (–3.00, 3.70)
LS Mean difference	−0.03 (0.88)					
95% CI	−1.77 to 1.70					
Eight-point SMBG, mmol/L						
Baseline	104	10.01 ± 3.30	9.46 (7.54, 11.72)	53	10.00 ± 2.98	9.66 (7.51, 11.83)
Week 24	94	10.00 ± 3.10	9.46 (7.75, 11.85)	45	9.42 ± 2.74	8.86 (7.91, 10.81)
Change^b^	92	0.28 ± 4.39	0.28 (–2.30, 2.84)	44	−0.58 ± 3.99	−0.59 (–2.96, 1.99)
Week 24 (LOCF)^c^	107	9.94 ± 3.00	9.49 (7.75, 11.85)	54	9.75 ± 2.83	9.02 (7.98, 11.25)
Change (LOCF)^b^	104	0.01 ± 4.30	0.01 (–2.58, 2.44)	53	−0.28 ± 3.92	−0.21 (–2.87, 2.69)
LS Mean difference	0.31 (0.50)					
95% CI	−0.68 to 1.30					

^a^The analysis excluded measurements obtained >14 days after treatment cessation

^b^Change from Baseline to Week 24

^c^For Week 24 (LOCF), the analysis included measurements obtained ≤14 days after the last dose of study medication

^d﻿^Analysis of number (%) of patients with HbA1c <7.5% (<58.5 mmol/mol) at Week 24 was not statistically significant; *P* = 0.6594﻿

^e^The analysis excluded measurements obtained >1 day after treatment cessation

^f^Nocturnal blood glucose was measured at 03:00 a.m.

*LS* least square, *Q1* quartile 1, *Q3* quartile 3 *SD*, standard deviation, *SMBG* self-monitored blood glucose

**Fig. 2:**
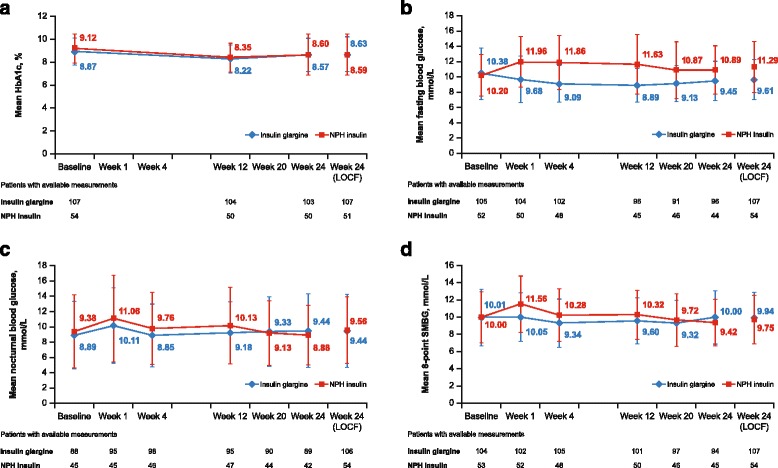
Variation in study variables over the 24-week study window for the mITT population. **a** Mean HbA1c (%). The analysis excluded measurements obtained >14 days after treatment cessation and for Week 24 (LOCF), the analysis included measurements obtained ≤14 days after the last dose of study medication. **b** Mean fasting blood glucose (mmol/l). The analysis excluded measurements obtained >1 day after treatment cessation. **c** Mean nocturnal blood glucose (mmol/l). The analysis excluded measurements obtained >1 day after treatment cessation. **d** Mean eight-point SMBG (mmol/l). Error bars represent the standard deviation. LOCF, last observation carried forward; SMBG, self-measured blood glucose

The percentage of patients reaching HbA1c <7.5% (<58.5 mmol/mol) at Week 24 was 18.7% in the insulin glargine group and 21.6%in the NPH insulin group, respectively. At all study time points and in both treatment groups,the mean HbA1c was numerically lower than that at baseline (Fig. 2a).

#### Secondary endpoints

From baseline to Week 24, the mean FBG decreased in patients who received insulin glargine from 10.38 ± 3.38 mmol/l to 9.61 ± 2.63 mmol/l and increased in patients who received NPH insulin from 10.20 ± 2.75 mmol/l to 11.29 ± 3.35 mmol/l (Table 1). There was considerable difference in change in FBG between the insulin glargine and NPH treatment groups (–0.76 ± 3.56 mmol/l versus 1.07 ± 3.64 mmol/l;95% CI: –2.62 to –0.76) and FBG was better controlled with insulin glargine than with NPH insulin at all study time points (see Fig. 2b).

The mean changes in nocturnal blood glucose and eight-point SMBG levels from baseline to Week 24 in the insulin glargine and NPH insulin groups were similar (Table 1). LBGI and HBGI that were calculated using the SMBG profile data as part of the posthoc analysis were also similar for both treatment groups (Additional file 3: Table S2). Furthermore, a trend for greater stability of mean nocturnal blood glucose and eight-point SMBG over the study period was observed in patients who received insulin glargine versus NPH insulin (Fig. 2c, d).

At baseline, the mean daily total and daily basal insulin doses for the insulin glargine and NPH insulin groups were numerically similar (0.84 ± 0.23units/kg/day versus 0.87 ± 0.29units/kg/day for total insulin and 0.29 ± 0.09units/kg/day versus 0.31 ± 0.13units/kg/day for basal insulin, respectively). Compared with baseline, at Week 24 the NPH insulin group had a greater numerical increase in both the mean daily total and basal insulin doses than the insulin glargine group (0.07 ± 0.16units/kg/day versus 0.19 ± 0.26 units/kg/day for total insulin and 0.02 ± 0.07units/kg/day versus0.11 ± 0.14units/kg/day for basal insulin, respectively; ANCOVA, Table 2). Consequently, at Week 24,the insulin glargine group had lower total and basal insulin doses than the NPH group (0.91 ± 0.25 units/kg/day versus 1.06 ± 0.32units/kg/day for total insulin, and 0.31 ± 0.09units/kg/day versus 0.43 ± 0.16units/kg/day for basal insulin, respectively). The mean changes in daily bolus insulin from baseline to Week 24 were numerically similar in the treatment groups (0.05 ± 0.12units/kg/day versus0.06 ± 0.17units/kg/day for the insulin glargine and NPH insulin groups, respectively).

**Table 2:** Daily insulin doses for the modified intention-to-treat analysis population with last observation carried forward for missing observations at Week 24

	Insulin glargine			NPH insulin		
	*n*	Mean ± SD	Median	*n*	Mean ± SD	Median
			(Q1, Q3)			(Q1, Q3)
Mean daily total insulin, units/kg/day ± SD^a^						
Baseline	107	0.84 ± 0.23	0.82 (0.67, 1.00)	53	0.87 ± 0.29	0.82 (0.70, 1.05)
Week 24	106	0.91 ± 0.25	0.91 (0.73, 1.04)	50	1.08 ± 0.32	1.11 (0.86, 1.28)
Change^b^	106	0.08 ± 0.16	0.07 (–0.02, 0.17)	50	0.19 ± 0.26	0.18 (–0.00, 0.35)
Week 24 (LOCF)	107	0.91 ± 0.25	0.91 (0.73, 1.04)	53	1.06 ± 0.32	1.06 (0.85, 1.26)
Change (LOCF)^b^	107	0.07 ± 0.16	0.07 (–0.03, 0.17)	53	0.19 ± 0.26	0.16 (–0.00, 0.35)
LS Mean Difference	−0.13 (0.03)					
95% CI	−0.19 to –0.06					
Mean daily basal insulin, units/kg/day ± SD^a^						
Baseline	107	0.29 ± 0.09	0.28 (0.23, 0.34)	53	0.31 ± 0.13	0.32 (0.23, 0.38)
Week 24	106	0.31 ± 0.09	0.29 (0.25, 0.37)	50	0.44 ± 0.16	0.44 (0.34, 0.52)
Change^b^	106	0.03 ± 0.07	0.02 (–0.01, 0.06)	50	0.12 ± 0.14	0.11 (0.04, 0.20)
Week 24 (LOCF)	107	0.31 ± 0.09	0.29 (0.25, 0.38)	53	0.43 ± 0.16	0.43 (0.30, 0.50)
Change (LOCF)^b^	107	0.02 ± 0.07	0.02 (–0.01, 0.06)	53	0.11 ± 0.14	0.11 (0.04, 0.20)
LS Mean Difference	−0.01 (0.02)					
95% CI	−0.13 to –0.06					
Mean daily bolus insulin, units/kg/day ± SD						
Baseline	107	0.55 ± 0.18	0.53 (0.42, 0.68)	52	0.58 ± 0.23	0.54 (0.40, 0.73)
Week 24	106	0.60 ± 0.19	0.58 (0.48, 0.71)	49	0.64 ± 0.23	0.63 (0.46, 0.81)
Change^b^	106	0.05 ± 0.12	0.04 (–0.02, 0.10)	49	0.06 ± 0.17	0.06 (–0.07, 0.18)
Week 24 (LOCF)	107	0.60 ± 0.19	0.57 (0.47, 0.71)	52	0.63 ± 0.23	0.63 (0.45, 0.81)
Change (LOCF)^b^	107	0.05 ± 0.12	0.04 (–0.03, 0.10)	52	0.06 ± 0.17	0.06 (–0.06, 0.18)
LS Mean Difference	−0.02 (0.02)					
95% CI	−0.06 to 0.03					

^a^Calculated as the weekly average

^b^Change from baseline to Week 24

*LS* least square, *Q1* quartile 1, *Q3* quartile 3, *SD* standard deviation

### Safety

Overall, both study drugs were well tolerated and only one patient discontinued treatment due to an SAE (increased blood glucosein the NPH insulin group; Table 3). A lower total incidence of drug-related treatment-emergent adverse events (TEAEs) was reported for patients in the insulin glargine group, compared with the NPH insulin group;the most common drug-related TEAEs were hypoglycaemia (33.6% versus 40.7%) and weight gain (0.9% versus 1.9%). A total of 10 serious TEAEs were reported: four events in three patients in the insulin glargine group and six events in six patients in the NPH insulin group. Diabetic ketoacidosis was the most commonly reported serious TEAE.

**Table 3:** Summary of safety data

	Insulin glargine	NPH insulin
	(*n* = 107)	(*n* = 54)
Mean duration of study treatment, days ± SD	167.5 ± 9.6	157.6 ± 38.9
Treatment discontinuation, n (%)	1 (1)	4 (7.3)
Due to TEAE^a^, n (%)	0 (0)	1 (1.9)^b^
≥1 treatment-related TEAE^a^, n (%)	37 (34.6)	24 (44.4)
Hypoglycaemia	36 (33.6)	22 (40.7)
Overweight	1 (0.9%)	1 (1.9)
Dizziness	0 (0)	1 (1.9)
Injection site swelling	0 (0)	1 (1.9)
Hunger	0 (0)	1 (1.9)
Blood glucose increased	0 (0)	1 (1.9)
Serious TEAE,^ac^ n (%)	4 (2.8)	6 (11.1)
Diabetic ketoacidosis	2 (1.9)	3 (5.6)
Mumps	1 (0.9)	0 (0)
Acute pancreatitis	1 (0.9)	0 (0)
Blood glucose increase	0 (0)	1 (1.9)
Hypoglycaemia	0 (0)	1 (1.9)
Respiratory tract infection	0 (0)	1 (1.9)
AGA-positive, n/n (%)		
Screening	74/107 (69.2)	43/55 (78.2)
Week 24	69/106 (65.1)	39/50 (78.0)
Shift from negative to positive, n/n (%)	7/32 (21.9)	5/10 (50.0)
Shift from positive to negative, n/n (%)	12/74 (16.2)	6/40 (15.0)
AGA titre, mean ± SD		
Screening	41.4 ± 90.2	41.3 ± 86.9
Week 24	56.6 ± 150.9	28.8 ± 44.3
AIA-positive, n (%)		
Baseline	65/107 (60.7)	38/55 (69.1)
Week 24	60/106 (56.6)	38/50 (76.0)
Shift from negative to positive, n/n (%)	5/41 (12.2)	5/14 (35.7)
Shift from positive to negative, n/n (%)	10/65 (15.4)	2/35 (5.7)
AIA titre, mean ± SD		
Screening	69.2 ± 177.1	50.1 ± 63.9
Week 24	66.9 ± 169.0	33.7 ± 34.0

^a^The TEAEs and serious TEAEs during the on-treatment period were presented by preferred terms

^b^Blood glucose increase

^c^Patients may have experienced more than one adverse event

*TEAE* treatment emergent adverse event, *AGA* anti-glargine antibody, *AIA* anti-insulin antibody, *SD* standard deviation

From screening to Week 24, the proportion of AGA- and AIA-positive patients in the insulin glargine group decreased by 4.1% for both AGA and AIA, respectively (Table 3). In the NPH insulin group, the proportion of AIA-positive patients increased by 6.9%, while the proportion of AGA-positive patients did not change.

The frequency and event rates of hypoglycaemia are summarised in Table 4. More than 90% of patients in both treatment groups experienced ≥1 episode of hypoglycaemia. However, the insulin glargine group had a numerically lower rate of any hypoglycaemic events per patient year than the NPH insulin group (68.6 ± 69.4 versus 84.6 ± 79.3). The proportion of patients who experienced ≥1 symptomatic hypoglycaemic event and the rate of symptomatic hypoglycaemic events per patient year were also numerically lower in the insulin glargine group than in the NPH insulin group. Only a small proportion of all patients experienced severe symptomatic hypoglycaemia (<2%). Nocturnal symptomatic hypoglycaemia was less common in patients who received insulin glargine (37.4%) than in those who received NPH insulin (46.3%).

**Table 4:** Summary of hypoglycaemia frequency and event rates

	Insulin glargine	NPH insulin
	(*n* = 107)	(*n* = 54)
Any hypoglycaemia^a^, n (%)	99 (92.5)	51 (94.4)
Events per patient year^b^, mean ± SD	68.6 ± 69.4	84.6 ± 79.3
Asymptomatic hypoglycaemia^a^, n (%)	93 (86.9)	47 (87.0)
Events per patient year^b^, mean ± SD	44.4 ± 48.7	52.3 ± 65.3
Symptomatic hypoglycaemia^a^, n (%)	74 (69.2)	41 (75.9)
Events per patient year^b^, mean ± SD	24.3 ± 45.8	32.3 ± 43.2
Severe symptomatic hypoglycaemia^a^, n (%)	1 (0.9)	1 (1.9)
Events per patient year^b^, mean ± SD	0.02 ± 0.20	0.04 ± 0.31
Nocturnal hypoglycaemia^a^, n (%)	83 (77.6)	42 (77.8)
Events per patient year^b^, mean ± SD	13.0 ± 15.0	14.2 ± 16.9
Nocturnal symptomatic hypoglycaemia^a^, n (%)	40 (37.4)	25 (46.3)
Events per patient year^b^, mean ± SD	3.6 ± 7.3	4.5 ± 7.4

^a^Calculated as the total number of patients with at least one episode from the first dose of treatment up to 24 h after the last dose of treatment divided by the total number of patients in the safety population

^b^Calculated as the total number of episodes divided by the total duration from the first dose to ≤24 h after the last dose of study medication (in years)

No accumulation of insulin glargine or insulin glargine metabolites M1 orM2 occurred after repeated dosing.

## Discussion

Insulin glargine, a long-acting insulin analogue, is available as a treatment option for paediatric patients with T1DM in Europe and the USA. However,as there are currently no data on the safety and efficacy of insulin glargine in Chinese children, it is not an approved treatment in China for T1DM in paediatric patients. To the authors’ knowledge, this is the first Phase III study investigating insulin glargine in Chinese paediatric patients with T1DM.

The data in this study show that Chinese paediatric patients with T1DM can achieve adequate glycaemic control with a once-daily administration of insulin glargine. The mean and median absolute changes in HbA1c were –0.25 ± 1.68%(–2.69 ± 18.32 mmol/mol) and –0.50% (–1.20, 0.60) (–5.46 mmol/mol [–13.11, 6.56]) after the 24-week treatment period with insulin glargine. The marketed and widely prescribed intermediate-acting NPH insulin served as reference treatment, and the mean and median absolute changes in HbA1c from baseline to Week 24 in the NPH insulin treatment group were –0.54 ± 1.67% (–5.55 ± 20.32 mmol/mol) and –0.40% (–1.20, 0.40) (–3.28 mmol/mol [–15.30, 4.92]), respectively.

Furthermore, at Week 24, a HbA1c of <7.5% (<58.5 mmol/mol) was achieved by 18.7 and 21.6% of patients in the insulin glargine and NPH insulin groups, respectively. Interestingly, in addition to the comparable effects that were observed on HbA1c, HBGI was also found to be similar between the treatment groups. This is consistent with a previous study, which found a strong correlation between HBGI and glycosylated haemoglobin levels [18].

Earlier studies involving the treatment of paediatric patients with T1DM with insulin glargine have reported reductions in HbA1c between –0.6 and 0.28%, comparable with the findings in our study [10, 11, 19–21]. A post-hoc analysis of data from the present study showed that the majority of patients in the insulin glargine and NPH insulin treatment groups achieved HbA1c ≤9.0% (≤74.9 mmol/mol) at Week 24 (71% and 72.6%), and therefore achieved acceptable glycaemic control as defined by the Chinese Diabetes Society guidelines [17]. A study by Schoberet al.,identical to the present study in length and insulin type/dosing, also reported comparable changes in HbA1c from baseline to Week 24 for insulin glargine and NPH insulin, although, in contrast to the present study, the changes from baseline were positive for both types of insulin (0.28 and 0.27%; *p* = 0.93) [11].

Previous reports in paediatric patients with T1DM have also reported greater reductions in FBG with insulin glargine versus NPH insulin,despite demonstrating comparable efficacy in terms of HbA1c [10, 11, 19–21]. The changes in FBG from baseline to Week 24 observed in this study support these findings. There was a considerable difference in the change in FBG between the insulin glargine and NPH insulin groups (–0.76 ± 3.56 mmol/l versus 1.07 ± 3.64 mmol/l); mean FBG decreased in the insulin glargine group (from 10.38 ± 3.38 mmol/l to 9.61 ± 2.63 mmol/l) and increased in the NPH insulin group (from 10.20 ± 2.75 mmol/l to 11.29 ± 3.35 mmol/l). Interestingly, a relatively large study (*N* = 175), which compared insulin glargine plus insulin lisprowith NPH insulin or lenteinsulin plus insulin lispro, also reported better control of FBG from baseline to endpoint with insulin glargine compared with NPH insulin or lente insulin (–3.3 mg/dl versus 1.1 mg/dl), although this was not statistically significant (*p* = 0.6962) [19]. Overall,the results in paediatric patients are broadly similar to those reported in adults with T1DM, which consistently demonstrate greater reductions in FBG, although results regarding reductions in HbA1c for insulin glargine versus NPH insulin are conflicting [14].

At the end of the 24-week treatment period, patients in the insulin glargine group used considerably lower total and basal insulin doses than the NPH group (0.91 ± 0.25units/kg/day versus1.06 ± 0.32units/kg/day for total insulin, and 0.31 ± 0.09 units/kg/dayversus0.43 ± 0.16units/kg/day for basal insulin, respectively) to achieve glycaemic control. Adequate glycaemic control is central to reducing the long-term complications of diabetes, and it is likely that a dosing regimen which achieves glycaemic control with the least amount of total/basal insulin dose per day, will enable the achievement of greater control with fewer incidences of hypoglycaemia. In the present study, the insulin glargine group had a numerically lower rate of any hypoglycaemia per patient year than the NPH insulin group (68.6 ± 69.4 versus 84.6 ± 79.3). The incidence of hypoglycaemia observed in the present study is less than that previously reported in studies involving European children; the previously mentioned study by Schoberet al.reported a greater proportion of patients experiencing ≥1 episode of symptomatic hypoglycaemia (78.9% and 79.3%, versus 69.2% and 75.9% in the present study) and severe hypoglycaemia (23.0% and 28.6%, versus 0.9% and 1.9% in the present study) with both insulin glargine and NPH insulin [11]. This suggests that insulin glargine and NPH insulin may be associated with a lower frequency of symptomatic and severe hypoglycaemia in Chinese paediatric patients with T1DM compared with their European counterparts. In accordance with this hypothesis, LBGI, a known predictor of severe hypoglycaemia, was similarly low in patients treated with insulin glargine and NPH insulin in this study. Although data on patients’ lifestyles were not collected in the present study, differences in factors such as bedtime and diet may have contributed to this apparent difference in hypoglycaemia. However, given that the changes in HbA1c from baseline to Week 24 in the study by Schoberet al.[11] were different to those in the present study, and that insulin dose was not presented, this comparison of hypoglycaemia between both studies is limited. In addition, nocturnal hypoglycaemia was experienced by a smaller proportion (<30%) of patients in the study by Schoberet al. compared with the present study (~78%) [11].

Overall, both insulin glargine and NPH insulin were well tolerated, and no new safety signals were observed in Chinese paediatric patients with T1DM when compared with the safety profiles reported by large trials in European and American paediatric T1DM populations [10, 11, 19].

Taken together, data from this study supports the hypothesis of previous studies: insulin glargine100 U/ml once daily at bedtime may offer glycaemic control benefits over NPH insulin 100 U/ml once or twice daily, despite having a comparable effect on HbA1c [14].

Conducting a single or double blinded trial was not possible owing to the differences in dosing schedule between insulin glargine and NPH insulin. Assessment of the outcomes, however, was based on objectively collected data, namely, HbA1c evaluated by central laboratories blinded to the treatment as well as SMBG values. Anotherone of the limitations of this study arose from patients discontinuing treatment prematurely during the treatment period, resulting in outcome data not being reported for Week 24. Assessment of the primary and secondary endpoints for these patients was therefore conducted using their last post-baseline on-treatment measurement for the calculation of Week 24 (LOCF). Given that LOCF does not take into consideration whether a patient’s condition was improving or deteriorating at the time of dropout, the analysis may have incorrectly increased, decreased or stabilised patients’ glycaemic measures, thereby introducing bias. The primary limitation of the present study,however,was the reduction in enrolment targetsdue to difficulties in the recruitment of patients. The small sample size resulted in statistical comparisons that were underpowered. Nonetheless, our results are broadly similar to those of other studies that compared insulin glargine and NPH insulin. To the authors’ knowledge, this is the first Phase III trial of insulin glargine in Chinese paediatric patients with T1DM, and therefore the results of the present study should be of value to the academic community and prescribing physicians.

## Conclusion

The results of this study provide evidence that in Chinese paediatric patients with T1DM, insulin glargine provides glycaemic control consistent with that observed in previous studies in Europe and the US, and may be associated with a numerically lower incidence rate of hypoglycaemia compared with NPH insulin. Insulin glargine was well tolerated, with no new safety signals observed in this previously understudied Chinese T1DM population.

## Additional files

Additional file 1: Supplementary Methods and Results. (DOC 25 kb)
Additional file 2: Table S1. Patient demographics and baseline disease characteristics. (DOCX 15 kb)
Additional file 3: Table S2. Low blood glucose risk index (LBGI) and high blood glucose risk index (HBGI) of self-monitored 8-point glucose in the modified intention-to-treat analysis by visit between baseline and Week 24. (DOCX 15 kb)

## Abbreviations

AGA: Anti-glargine antibody
AIA: Anti-insulin antibody
FBG: Fasting blood glucose
HBGI: High blood glucose risk index
LBGI: Low blood glucose risk index
LOCF: Last observation carried forward
NPH: Neutral protamine Hagedorn
SAE: Severe adverse event
SMBG: Self-monitored blood glucose
TEAE: Treatment-emergent adverse events.
