# Supplementary material for: A randomised, open-labelstudy of insulin glargine or neutral protamine Hagedorn insulin in Chinese paediatric patients with type 1 diabetes mellitus
Source: BMC Endocr Disord. 2016 Nov 26;16:67. doi: 10.1186/s12902-016-0146-2 (PMC5124261; doi:10.1186/s12902-016-0146-2)
Supplement: Additional file 1: — Supplementary Methods and Results. (DOC 25 kb) [file 12902_2016_146_MOESM1_ESM.doc]

**Supplementary materials**

**Supplementary Methods**

*Patients and study design*

Key exclusion criteria included HbA1c <7% (<53.0 mmol/mol) or >12% (>107.7 mmol.mol) at screening, diabetes other than type 1 DM, use of oral or parenteral glucose-lowering medications other than insulin, and contraindications to any of the study medications as defined in the Chinese product labels.

Insulin aspart (NovoRapid, Novo Nordisk) solution for injection was provided as the sole source of bolus insulin. Insulin dose was adjusted individually to maintain the desired degree of metabolic control without hypoglycaemia, as defined by the following targets: FBG, 90–145 mg/dL (5.0–8.0 mmol/L), bedtime blood glucose 120–180 mg/dL (6.7–10.0 mmol/L), nocturnal blood glucose 80–162 mg/dL (4.4–9.0 mmol/L) and HbA1c <7.5% (<53.0 mmol/mol).

*Study objectives*

The original primary objective was to show non-inferiority of insulin glargine to NPH insulin for absolute change in HbA1c from baseline to Week 24. Howeverdue to the reductionin the enrolment target, and the primary objective was updated to descriptions of the efficacy and safety of insulin glargine in paediatric subjects with type 1 DM, using the primary endpoint of absolute change in HbA1c from baseline to Week 24.

Hypoglycaemia was classified as asymptomatic (blood glucose values <70 mg/dL [3.9 mmol/L] without clinical symptoms), symptomatic (blood glucose <70 mg/dL [3.9 mmol/L] with associated clinical symptoms), severe symptomatic (hypoglycaemia requiring the assistance of a third party or involving a seizure, coma, unconsciousness or the use of glucagon), and nocturnal (occurring between 23:00–07:00).

*Statistical methods*

In the original study protocol, a recruitment target of 366 subjects (insulin glargine, n = 244; NPH insulin, n = 122) was calculated to power the study to investigate non-inferiority of insulin glargine to NPH insulin for absolute change in HbA1c from baseline to Week 24. Following the protocol amendment, the enrolment target was reduced to 150 patients (insulin glargine, n = 100; NPH insulin, n = 50), and the primary analysis was amended to descriptive statistics for absolute change in HbA1c from baseline to Week 24, and all secondary endpoints.

Insulin glargine pharmacokinetics were analysed at selected study sites for randomised patients who received at least one dose of insulin glargine and provided an evaluable blood sample at Weeks 1, 2 or 4. The antibody analysis was performed for all randomised subjects who contributed at least one blood sample during screening or at Weeks 1 or 24.

**Supplementary Results**

*Pharmacokinetic profile*

Following repeated once-daily subcutaneous dosing of insulin glargine in a subset of 40 subjects, the M1 metabolite was the principal circulating insulin glargine compound detected in blood plasma. Within a time interval of 9.5 to 16.5 hours following the previous evening’s injection, mean plasma M1 concentrations were 0.855 ng/mL at Week 1, 0.760 ng/mL at Week 2 and 0.672 ng/mL at Week 4. Mean plasma concentrations of insulin glargine and insulin glargine metabolite M2 were below LLOQ (0.200 ng/mL). No accumulation of insulin glargine or insulin glargine metabolites M1 orM2 occurred after repeated dosing.
