# Supplementary material for: A randomised, open-labelstudy of insulin glargine or neutral protamine Hagedorn insulin in Chinese paediatric patients with type 1 diabetes mellitus
Source: BMC Endocr Disord. 2016 Nov 26;16:67. doi: 10.1186/s12902-016-0146-2 (PMC5124261; doi:10.1186/s12902-016-0146-2)
Supplement: Additional file 2: Table S1. — Patient demographics and baseline disease characteristics. (DOCX 15 kb) [file 12902_2016_146_MOESM2_ESM.docx]

**Supplementary Table 1**. Patient demographics and baseline disease characteristics

|  | Insulin glargine (n = 107) | NPH insulin (n = 54) |
| --- | --- | --- |
| Mean age, years ± SD | 12.3 ± 3.2 | 12.2 ± 3.5 |
| Age group, years, n (%)  <12  ≥12 | 42 (39.3)  65 (60.7) | 22 (40.7)  32 (59.3) |
| Male, n (%) | 44 (41.4) | 19 (35.2) |
| Mean height, cm ± SD | 149.2 ± 16.4 | 148.5 ± 15.8 |
| Mean BMI, kg/m^2^ ± SD | 18.7 ± 2.9 | 18.2 ± 2.6 |
| Mean HbA1c level, % ± SD | 8.87 ± 1.21 | 9.12 ± 1.29 |
| HbA1c category, n (%)  <9%  ≥9% | 48(44.9)  59(55.1) | 23 (42.6)  31 (57.4) |
| Tanner puberty stage, n (%)  Stage 1  Stage 2  Stage 3  Stage 4  Stage 5 | 30 (28.0)  14 (13.1)  16 (15.0)  33 (30.8)  14 (13.1) | 12 (22.2)  10 (18.5)  12 (22.2)  16 (29.6)  4 (7.4) |
| Mean duration of diabetes, years ± SD | 3.83 ±2.93 | 3.55 ± 2.25 |
| Mean prior insulin dose, units/day ± SD*  Basal insulin  Bolus insulin  Total insulin | 12.35 ± 5.79  23.29 ± 10.28  35.47 ± 14.86 | 12.89 ± 6.13  23.61 ± 11.21  35.81 ± 15.22 |
| Diabetic retinopathy, n (%) | 0 (0) | 0 (0) |
| Diabetic sensory or motor neuropathy, n (%) | 0 (0) | 0 (0) |
| Diabetic nephropathy, n (%)  Microalbuminurea | 0  0 | 1 (1.9)†  1 (100) |

*Baseline insulin doses were calculated using the average doses administered on the 7 days after the first study drug administration

†Percentages are calculated using the number of patients with diabetic nephropathy = 'yes' as denominator

SD, standard deviation.
