# Supplementary material for: A randomised, open-labelstudy of insulin glargine or neutral protamine Hagedorn insulin in Chinese paediatric patients with type 1 diabetes mellitus
Source: BMC Endocr Disord. 2016 Nov 26;16:67. doi: 10.1186/s12902-016-0146-2 (PMC5124261; doi:10.1186/s12902-016-0146-2)
Supplement: Additional file 3: Table S2. — Low blood glucose risk index (LBGI) and high blood glucose risk index (HBGI) of self-monitored 8-point glucose in the modified intention-to-treat analysis by visit between baseline and Week 24. (DOCX 15 kb) [file 12902_2016_146_MOESM3_ESM.docx]

**Supplementary Table 2.** Low blood glucose risk index (LBGI) and high blood glucose risk index (HBGI) of self-monitored 8-point glucose in the modified intention-to-treat analysis by visit between baseline and Week 24

|  |  | Insulin glargine  (n = 107) | |  | NPH insulin  (n = 54) | |
| --- | --- | --- | --- | --- | --- | --- |
|  | n | Mean ± SD | Median  (min, max) | n | Mean ± SD | Median  (min, max) |
| LBGI  Baseline  Week 1  Week 4  Week 12  Week 20  Week 24  Week 24 (LOCF) | 104  102  105  101  97  94  107 | 1.75 ± 2.76  1.17 ± 1.79  1.40 ± 1.84  1.32 ± 2.05  1.36 ± 2.12  1.05 ± 1.71  1.09 ± 1.74 | 0.55 (0.0, 18.3)  0.42 (0.0, 9.8)  0.62 (0.0, 7.6)  0.45 (0.0, 9.1)  0.64 (0.0, 9.8)  0.27 (0.0, 9.0)  0.31 (0.0, 9.0) | 53  52  48  50  46  45  54 | 1.59 ± 1.90  1.40 ± 2.79  1.54 ± 2.22  1.16 ± 1.74  0.96 ± 1.21  1.51 ± 2.30  1.33 ± 2.14 | 0.84 (0.0, 7.4)  0.20 (0.0, 14.0)  0.97 (0.0, 12.2)  0.41 (0.0, 6.6)  0.47 (0.0, 5.0)  0.69 (0.0, 10.8)  0.49 (0.0, 10.5) |
| HBGI  Baseline  Week 1  Week 4  Week 12  Week 20  Week 24  Week 24 (LOCF) | 104  102  105  101  97  94  107 | 12.35 ± 10.49  11.93 ± 9.37  9.77 ± 8.96  10.40 ± 7.94  9.47 ± 8.49  11.63 ± 10.32  11.27 ± 9.29 | 9.57 (0.0, 43.5)  10.24 (0.1, 38.5)  6.72 (0.1, 47.4)  9.02 (0.0, 30.4)  7.48 (0.0, 47.5)  9.17 (0.0, 57.6)  9.38 (0.0, 53.4) | 53  52  48  50  46  45  54 | 12.09 ± 9.52  17.17 ± 11.03  12.85 ± 10.07  12.73 ± 8.78  10.79 ± 10.08  9.85 ± 8.63  10.83 ± 8.67 | 10.57 (0.5, 53.7)  15.46 (1.0, 47.8)  11.80 (0.0, 57.6)  12.37 (0.0, 39.9)  8.15 (0.1, 48.5)  7.24 (0.0, 40.6)  8.69 (0.6, 40.6) |
